# Evaluation of heart rate variability using 24‐hour Holter electrocardiography in hypertensive patients

**DOI:** 10.1002/joa3.12469

**Published:** 2020-11-28

**Authors:** Rerdin Julario, Eka Prasetya Budi Mulia, Dita Aulia Rachmi, Maya Qurota A’yun, Imanita Septianda, Ivana Purnama Dewi, Rahima Ratna Juwita, Budi Baktijasa Dharmadjati

**Affiliations:** ^1^ Department of Cardiology and Vascular Medicine Faculty of Medicine Universitas Airlangga ‐ Dr. Soetomo General Hospital Surabaya Indonesia

**Keywords:** autonomic nervous system, blood pressure, heart rate variability, Holter, hypertension

## Abstract

**Background:**

Hypertension (HTN) remains a serious risk factor for cardiovascular mortality across the world. Hypertensive state has been shown to be associated with autonomic nervous function. This study aimed to explore the association between autonomic nervous impairment assessed by heart rate variability (HRV) and HTN.

**Methods:**

A total of 52 hypertensive and 55 non‐hypertensive patients were consecutively studied using 24‐hour Holter. The hypertensive patients were grouped into controlled blood pressure (BP) and uncontrolled BP. This study compared HRV in non‐hypertensive and hypertensive patients; and hypertensive patients with controlled and uncontrolled BP. HRV parameters include time and frequency domain.

**Results:**

Mean age for hypertensive and non‐hypertensive patients were 53.58 ± 14.31 and 44.89 ± 16.63 years old, respectively. Median (IQR) SDNN for hypertensive and non‐hypertensive group were 109.00 (90.00‐145.00) and 129.00 (107.00‐169.00), respectively. SDNN, ASDNN, rMSSD, pNN50, BB50, VLF, and HF values were significantly lower in the hypertensive group compared to non‐hypertensive group (all *P* < .05). A multiple regression analysis showed that HRV parameters: SDANN, ASDNN, rMSSD, and LF values were independent risk factors of HTN. SDNN, SDANN, ASDNN, VLF, LF, and HF values were significantly lower in the uncontrolled BP compared to controlled BP group (all *P* < .05). A multiple regression analysis showed that HRV parameters: SDNN, SDANN, rMSSD, and HF values were independent risk factors of uncontrolled BP in hypertensive patients.

**Conclusions:**

Our study showed that cardiac autonomic nervous impairment, as demonstrated by reduced HRV, is significantly associated with HTN. Decreased HRV was more evident in uncontrolled BP than in controlled BP group.

## Graphical abstract

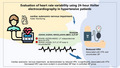

## 1 INTRODUCTION

Hypertension (HTN) remains a serious risk factor for cardiovascular mortality across the world.^1^ The asymptomatic nature of HTN may hinder diagnosis and prompt initiation of appropriate therapies.^2^ Essential hypertensive disorders can be identified not only by reduced parasympathetic tones but also by a severe sympathetic overdrive, resulting in an increase in resting heart rate values.^3^, ^4^ Another literature summarises sympathetic dysregulation in the differentiation risk in stages of HTN (mild, moderate, severe), form of hypertension in young, middle‐aged, and elderly, white‐coat HTN, masked HTN, and gestational HTN.^5^ Heart rate variability (HRV) influences the autonomic control of cardiac function. HRV reflects the autonomous nervous system response to external stimuli. Abnormal HRV represented autonomous imbalance and was associated with worse cardiovascular outcome.^6^

One of the major studies that found reduced HRV in males and females with systemic HTN was the Framingham Heart Study. This study also found that LF (low frequency) power of HRV was correlated with new‐onset HTN in men. The assessment of HRV using 24‐hour Holter Electrocardiography (ECG) is a simple and reliable tool to assess autonomic imbalance in HTN patients.^7^ Analyzing HRV may be beneficial in improving our understanding of underlying pathophysiology, optimizing treatment modalities for hypertensive patients subsets with signs of autonomic impairment, and predicting future major adverse cardiovascular events (MACE) in patients at risk.^3^ This study aimed to explore the association between HRV in non‐hypertensive and hypertensive patients and between controlled and uncontrolled BP of hypertensive patients.

## 2 METHODS

### 2.1 Study design and study setting

This was an observational study using a retrospective cross‐sectional design. This study was held at Cardiac Center—Dr Soetomo General Hospital, Surabaya, Indonesia.

### 2.2 Study population

A total of 52 patients with HTN or hypertensive heart disease and 55 patients without HTN or hypertensive heart disease as controls in 24‐hour Holter ECG Registry Data from April 2019 to March 2020 were chosen and included in this study. All subjects are in the ages between 15 and 80 years old. All hypertensive patients received antihypertensive medication. Patients with hypertension were grouped into controlled blood pressure (BP) (n = 18), and uncontrolled BP (n = 34). BP of each subjects were measured right before Holter recording at outpatient clinic. Patients with second or third‐degree atrioventricular block, atrial fibrillation, atrial flutter, sinus arrest, pacemaker implantation, pregnant, and missing required data were excluded.

Blood pressure was recorded with validated digital BP device Omron M3 (HEM‐7200‐E) in sitting posture after five‐minutes of rest. The diagnosing criteria of HTN were BP ≥140/90 mm Hg (according to Joint National Committee/JNC VII classification). Subject with BP ranging from 100‐139/60‐89 mm Hg was recruited into non‐hypertensive group. Subject with BP ≥140/90 mm Hg or self‐reported use of anti‐hypertensive drugs during the 2 weeks prior to the clinical examination was recruited into hypertensive group. Subject with self‐reported use of anti‐hypertensive drugs during the 2 weeks prior to the clinical examination and BP ≥140/90 mm Hg was recruited into uncontrolled BP group.

### 2.3 Ethical clearance

Institutional committee of research and ethics of Dr Soetomo General Academic Hospital gave ethical clearance and approved the study (Ref: 1822/KEPK/II/2020).

### 2.4 Data collection

All participants were subjected to 24‐hour Holter ECG monitoring (MARS PC Holter Monitoring and Review System software and SEER Light Digital Holter Recorder; GE). Each patient was given a detailed explanation of how the test was done and how to handle the recorder. Holter ECG was placed in the patient's waist, and the electrode leads were placed appropriately on the chest. The patients were instructed to go home, recommence normal daily activities, and return to hospital after 24 hours.

### 2.5 HRV analysis

Data analysis of quantitative HRV was carried out based on the guidelines of the European Society of Cardiology and the North American Society of Pacing and Electrophysiology.^8^ HRV parameters, including time domain and frequency domain, were obtained from 24‐hour Holter monitoring. We used seven time‐domain variables: average of all intervals between normal beats excluding ectopy or noise intervals (mean NN), standard deviation of intervals of all normal beat (SDNN), standard deviation of five‐minute mean R‐R interval (SDANN), mean of five‐minute standard deviations of intervals (ASDNN), root mean square of the difference of successive R‐R intervals (rMSSD), percentage of intervals that are more than 50 ms different from the previous interval (pNN50), count of intervals that are more than 50 ms different from the previous interval (BB50). Four frequency domain variables included very low frequency (VLF: 0.0033‐0.04 Hz), low frequency (LF: 0.04‐0.15 Hz), high frequency (HF:0.15‐0.4 Hz), and low‐frequency/high‐frequency ratio (LF/HF). Fast Fourier transform was used in this analysis.

### 2.6 Statistical analysis

The statistical analysis was done using SPSS version 25 software for Windows (IBM Corp). Descriptive statistics of continuous data were given as mean (standard deviations [SD]) or median (interquartile range [IQR]) depend on data distribution, while categorical data were given as n (%). Data distribution was tested using One‐Sample Kolmogorov–Smirnov test. Continuous variables with normal distribution were analyzed by an independent T‐test. Non‐normal distributed data were analyzed by Mann–Whitney U test. Chi‐square test was used to compare categorical variables. The HRV parameters as risk factors for HTN and uncontrolled BP of hypertensive patients were determined by multivariate logistic regression model after adjusting for age, sex, body mass index (BMI), amiodarone use, beta‐blockers use, of angiotensin‐converting enzyme inhibitors/ angiotensin II receptor blockers (ACE‐i/ARB) use, calcium channel blocker (CCB) use, and diuretic use. Receiver Operating Characteristic (ROC) curve was used to determine the optimal cut‐off of HRV parameters. *P* values less than .05 were considered statistically significant.

## 3 RESULTS

### 3.1 Baseline characteristics of study population

Characteristics of study population are summarized in Table 1. The study involved a total of 107 patients: 52 hypertensive patients (28 males) and 55 non‐hypertensive patients (24 males). There was no difference in distribution of sex between two groups. Mean age for hypertensive and non‐hypertensive patients were 53.58 ± 14.31 and 44.89 ± 16.63 years old, respectively. Mean heart rate for hypertensive and non‐hypertensive patients were 76.67 ± 14.03 and 74.27 ± 12.49 bpm, respectively. Diabetes mellitus (DM) was found in 3.8% of hypertensive patients and 3.6% of non‐hypertensive patients. History of arrhythmia became the most common comorbidity in both groups: 50.0% in hypertensive patients and 81.8% in non‐hypertensive patients. The use of ACE‐i/ARB and CCB was higher in hypertensive group, while the use of anti‐arrhythmic drug amiodarone was higher in non‐hypertensive group.

**TABLE 1:** Baseline characteristics of study population

Variables	HTN (N = 52)	Non‐HTN (N = 55)	*P*
Age (y)	53.58 ± 14.31	44.89 ± 16.63	.005
Sex male (n, %)	28 (53.8%)	24 (43.6%)	.291
BMI (kg/m^2^)	26.70 ± 3.63	25.76 ± 3.37	.165
Heart rate (bpm)	76.67 ± 14.03	74.27 ± 12.49	.351
SBP (mm Hg)	140.00 (120.00‐157.25)	120.00 (110.00‐125.00)	<.001
DBP (mm Hg)	90.00 (80.00‐100.00)	80.00 (75.00‐81.00)	<.001
Stroke (n, %)	1 (1.9%)	0 (0.0%)	.486
History of arrhythmia (n, %)	26 (50.0%)	45 (81.8%)	<.001
DM (n, %)	2 (3.8%)	2 (3.6%)	.954
VHD (n, %)	2 (3.8%)	0 (0.0%)	.234
CAD (n, %)	12 (23.1%)	5 (9.1%)	.048
CHD (n, %)	0 (0.0%)	3 (5.5%)	.244
Medication:			
Beta blocker (n, %)	34 (65.4%)	30 (54.5%)	.253
Amiodaron (n, %)	5 (9.6%)	12 (21.8%)	.084
CCB (n, %)	14 (26.9%)	5 (9.1%)	.016
ACE‐i/ARB (n, %)	28 (53.8%)	12 (21.8%)	.001
Diuretic (n, %)	8 (15.4%)	4 (7.3%)	.228

Note

Data are presented as mean ± SD, median (IQR), n (%).

Abbreviations: ACE‐I, angiotensin‐converting enzyme inhibitors; ARB, angiotensin II receptor blockers; BMI, body mass index; CAD, coronary artery disease; CCB, calcium channel blocker; CHD, congenital heart disease; DBP, diastolic blood pressure; DM, diabetes mellitus; HTN, hypertension; SBP, systolic blood pressure; VHD, valvular heart disease.

Table 2 showed baseline characteristics of hypertensive population with controlled and uncontrolled BP. The mean age for controlled and uncontrolled BP patients were 50.06 ± 13.97 and 55.44 ± 14.34 years old, respectively. Comorbidities and medications were evently distributed between two groups.

**TABLE 2:** Baseline characteristics of hypertensive population with controlled and uncontrolled BP

Variables	Uncontrolled BP (N = 34)	Controlled BP (N = 18)	*P*
Age (y)	55.44 ± 14.34	50.06 ± 13.97	.200
Sex male (n, %)	20 (58.8%)	8 (44.4%)	.322
BMI (kg/m^2^)	27.41 ± 3.24	25.38 ± 4.04	.054
Heart rate (bpm)	77.26 ± 12.25	75.56 ± 17.24	.712
SBP (mm Hg)	151.50 (140.00‐165.00)	115.00 (110.00‐120.00)	<.001
DBP (mm Hg)	95.00 (90.00‐100.00)	80.00 (73.75‐80.00)	<.001
Stroke (n, %)	1 (2.9%)	0 (0.0%)	.463
History of arrhythmia (n, %)	19 (55.9%)	7 (38.9%)	.244
DM (n, %)	1 (2.9%)	1 (5.6%)	.641
VHD (n, %)	0 (0.0%)	2 (11.1%)	.115
CAD (n, %)	8 (23.5%)	4 (22.4%)	.915
CHD (n, %)	0 (0.0%)	0 (0.0%)	‐
Medication:			
Beta blocker (n, %)	22 (64.7%)	12 (66.7%)	.888
Amiodaron (n, %)	4 (11.8%)	1 (5.6%)	.648
CCB (n, %)	11 (32.4%)	3 (16.7%)	.329
ACE‐i/ARB (n, %)	19 (55.9)	9 (50.0%)	.686
Diuretic (n, %)	5 (14.7)	3 (16.7)	.852

Note

Data are presented as mean ± SD, median (IQR), n (%).

Abbreviations: ACE‐I, angiotensin‐converting enzyme inhibitors; ARB, angiotensin II receptor blockers; BMI, body mass index; CAD, coronary artery disease; CCB, calcium channel blocker; CHD, congenital heart disease; DBP, diastolic blood pressure; DM, diabetes mellitus; HTN, hypertension; SBP, systolic blood pressure; VHD, valvular heart disease.

### 3.2 Heart rate variability analysis in hypertensive and non‐hypertensive patients

**FIGURE 1:**
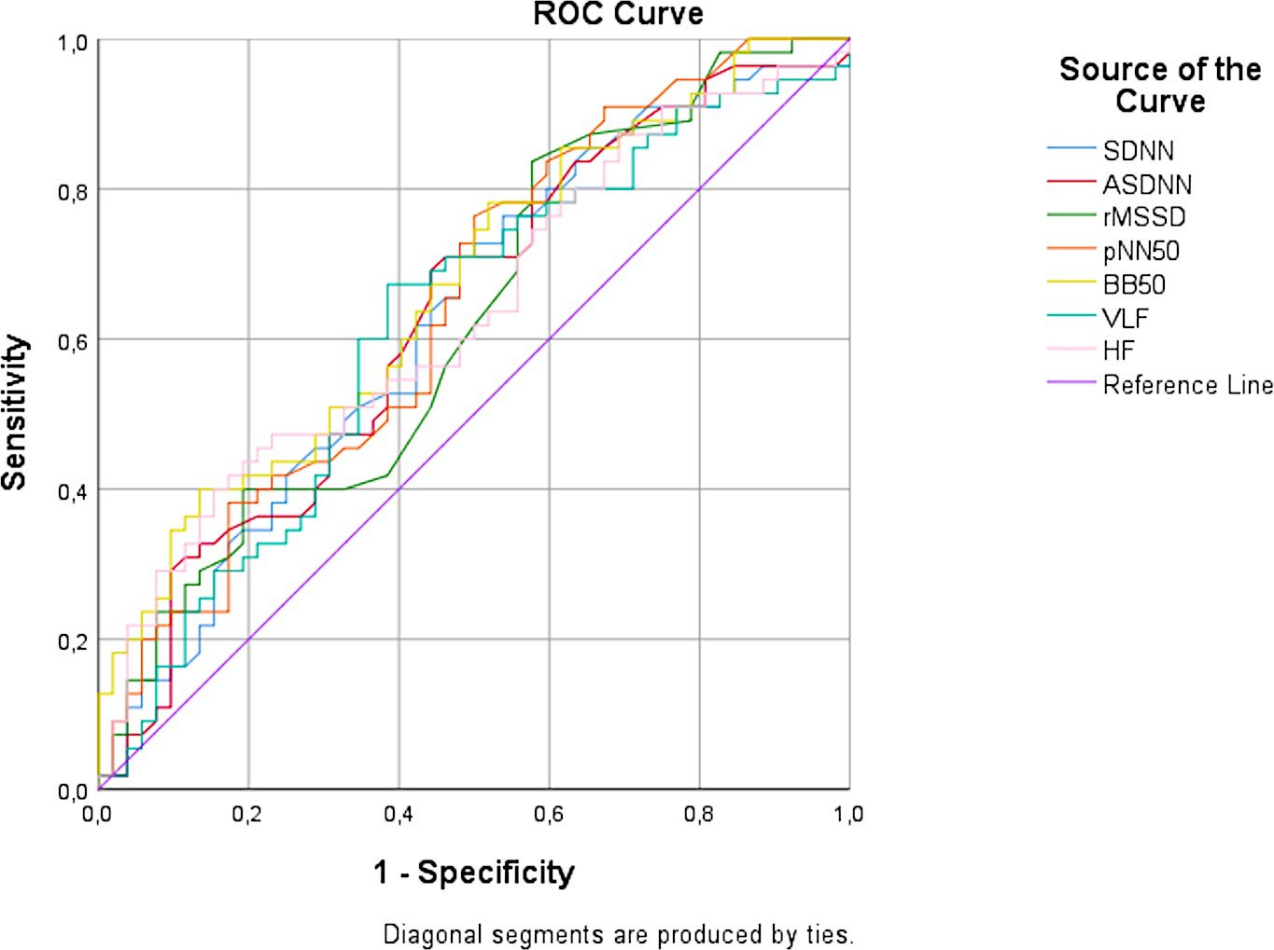
Receiver operator characteristic (ROC) curve for the prediction of hypertension by SDNN, ASDNN, rMSSD, pNN50, BB50, VLF, and HF. The 95% CIs of the area under the ROC curve are presented in Table S1

Table 3 shows the correlation between HRV parameters obtained from 24‐hour Holter ECG recordings and HTN. Median (IQR) SDNN for hypertensive and non‐hypertensive group were 109.00 (90.00‐145.00) and 129.00 (107.00‐169.00), respectively. SDNN, ASDNN, rMSSD, pNN50, BB50, VLF, and HF values were significantly lower in the hypertensive group compared to non‐hypertensive group (all *P* < .05). Based on the shortest distance on the ROC curve (corresponding to the largest sum of sensitivity and specificity) (Figure 1, Table S1), optimal cut‐off for SDNN was 111.5 ms (sensitivity 71% and specificity 52%, area under the curve/AUC 0.626), ASDNN was 39.5 ms (sensitivity 78% and specificity 43%, AUC 0.631), rMSSD was 20.5 ms (sensitivity 84% and specificity 43%, AUC 0.621), pNN50 was 36.5% (sensitivity 73% and specificity 52%, AUC 0.646), BB50 was 2913.50 beats (sensitivity 78% and specificity 48%, AUC 0.668), VLF was 23.57 ms (sensitivity 75% and specificity 46%, AUC 0.619), and HF was 9.07 ms (sensitivity 71% and specificity 44%, AUC 0.634).

**TABLE 3:** Comparison of heart rate variability parameters in hypertensive and non‐hypertensive patients

HRV parameters	HTN (N = 52)	Non‐HTN (N = 55)	*P*
Time domain:			
mean NN (ms)	746.00 (698.25‐853.00)	801.00 (730.00‐884.00)	.091
SDNN (ms)	109.00 (90.00‐145.00)	129.00 (107.00‐169.00)	.024
SDANN (ms)	101.00 (79.00‐144.75)	119.00 (90.00‐153.00)	.109
ASDNN (ms)	44.00 (34.25‐66.50)	54.00 (40.00‐81.00)	.020
rMSSD (ms)	27.50 (19.00‐34.50)	30.00 (25.00‐48.00)	.030
pNN50 (%)	29.00 (9.25‐90.75)	75.00 (27.00‐194.00)	.009
BB50 (beats)	3627.00 (1330.50‐8270.50)	6825.00 (3122.00‐15 530.00)	.003
Frequency domain:			
VLF (ms)	24.37 (19.31‐36.18)	29.78 (22.63‐44.76)	.034
LF (ms)	15.72 (11.90‐23.57)	19.99 (13.91‐35.44)	.053
HF (ms)	10.44 (6.47‐13.82)	12.94 (8.75‐19.14)	.017
LF/HF ratio	1.62 (1.35‐2.33)	1.49 (1.25‐2.12)	.336

Note

Data are presented as median (IQR).

Abbreviations: ASDNN, mean of five‐minute standard deviations of intervals; BB50, count of intervals that are more than 50 ms different from the previous interval; HF, high‐frequency power; HTN, hypertension; HRV, heart rate variability; LF, low‐frequency power; mean NN, average of all intervals between normal beats excluding ectopy or noise intervals; pNN50, percentage of intervals that are more than 50 ms different from the previous interval; rMSSD, root mean square of the difference of successive R‐R intervals; SDANN, standard deviation of five‐minute mean R‐R interval; SDNN, standard deviation of intervals of all normal beat.

### 3.3 Multivariate adjusted factors related to HTN

Multiple regression analysis showed that HRV parameters: SDANN, ASDNN, rMSSD, and LF values were independent risk factors of HTN after adjusting for age, sex, BMI, amiodarone use, beta‐blockers use, ACE‐i/ARB use, CCB use, and diuretic use (Table 4).

**TABLE 4:** Multivariate logistic regression for risk of hypertension

	B	S.E	Wald	*P*‐value	Exp (B)	95% CI lower limit	95% CI upper limit
Age	0.042	0.016	6.478	.011	1.043	1.010	1.077
Amiodaron use	−1.860	0.770	5.845	.016	0.156	0.034	0.703
ACE‐i/ARB use	1.613	0.507	10.111	.001	5.016	1.856	13.552
SDANN	−0.018	0.007	6.638	.010	0.982	0.969	0.996
ASDNN	0.070	0.026	7.082	.008	1.073	1.019	1.130
rMSSD	−0.048	0.019	6.210	.013	0.953	0.917	0.990
LF	−0.093	0.044	4.415	.036	0.911	0.835	0.994

Abbreviations: ACE‐I, angiotensin‐converting enzyme inhibitors; ARB, angiotensin II receptor blockers; ASDNN, mean of five‐minute standard deviations of intervals; LF, low‐frequency power; rMSSD, root mean square of the difference of successive R‐R intervals; SDANN, standard deviation of five‐minute mean R‐R interval.

### 3.4 Heart rate variability analysis in uncontrolled and controlled blood pressure in hypertensive patients

**FIGURE 2:**
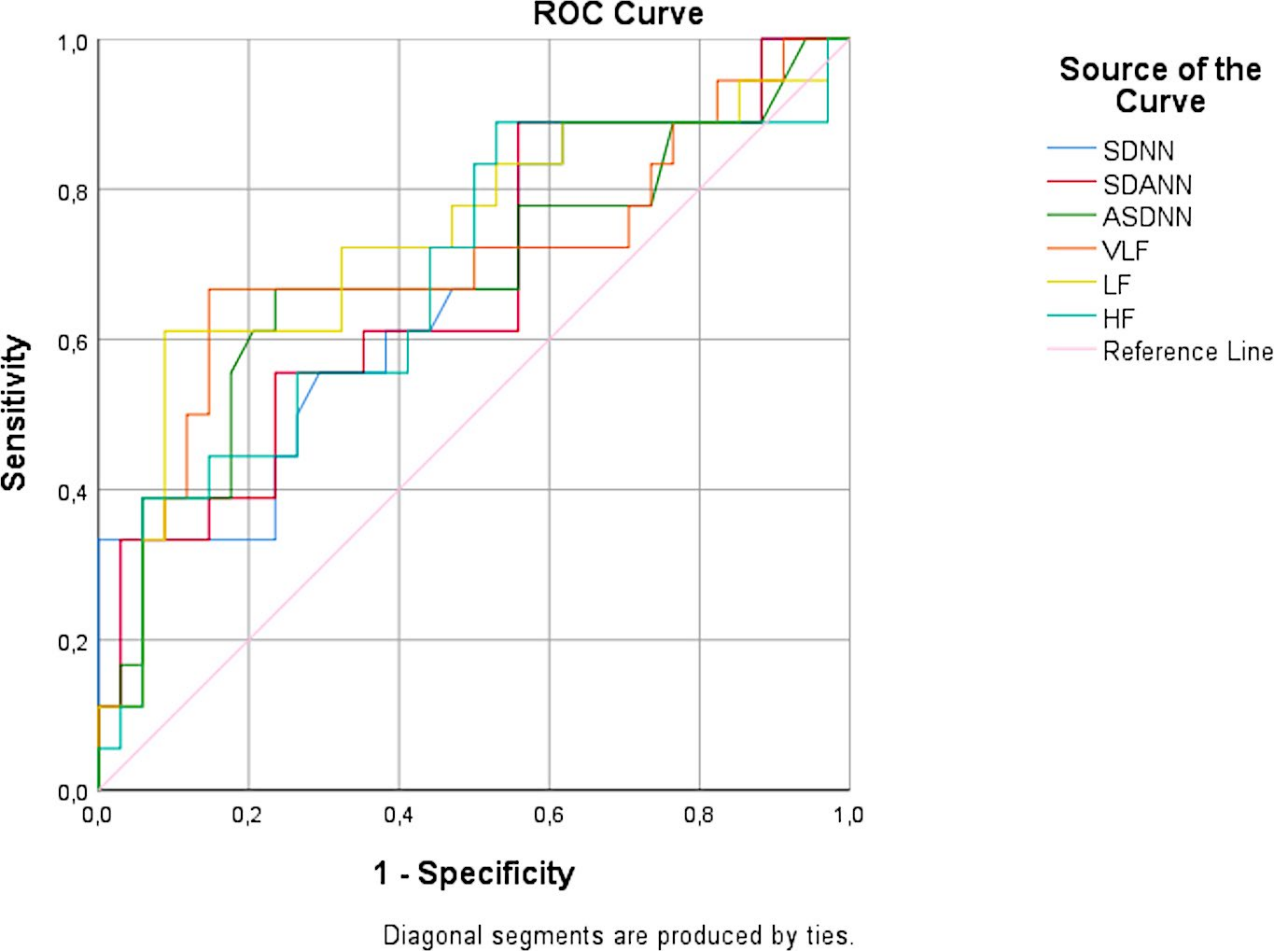
Receiver operator characteristic (ROC) curve for the prediction of uncontrolled blood pressure in hypertensive group by SDNN, SDANN, ASDNN, VLF, LF and HF. The 95% CIs of the area under the ROC curve are presented in Table S2

Table 5 shows the correlation between HRV parameters obtained from 24‐hour Holter ECG recordings and the control status of BP in hypertensive group. Median (IQR) SDNN for uncontrolled and controlled BP were 105.00 (89.00‐131.00) and 128.50 (99.00‐197.00), respectively. SDNN, SDANN, ASDNN, VLF, LF, and HF values were significantly lower in the uncontrolled BP group compared to controlled BP group (all *P* < .05). Based on the shortest distance on the ROC curve (corresponding to the largest sum of sensitivity and specificity) (Figure 2, Table S2), optimal cut‐off for SDNN was 96.50 ms (sensitivity 83% and specificity 44%, AUC 0.672), SDANN was 83.50 ms (sensitivity 89% and specificity 44%, AUC 0.673), ASDNN was 51.50 ms (sensitivity 67% and specificity 73%, AUC 0.685), VLF was 30.92 ms (sensitivity 67% and specificity 85%, AUC 0.698), LF was 23.03 ms (sensitivity 61% and specificity 91%, AUC 0.735), and HF was 7.71 ms (sensitivity 89% and specificity 44%, AUC 0.680).

**TABLE 5:** Comparison of component values of heart rate variability parameters in controlled and uncontrolled blood pressure in hypertensive group

HRV parameters	Uncontrolled HTN (N = 34)	Controlled HTN (N = 18)	*P*
Time domain:			
mean NN (ms)	754.50 (719.00‐853.00)	722.50 (666.00‐925.00)	.617
SDNN (ms)	105.00 (89.00‐131.00)	128.50 (99.00‐197.00)	.043
SDANN (ms)	98.50 (76.00‐117.00)	125.00 (91.00‐181.00)	.041
ASDNN (ms)	42.50 (34.00‐49.00)	61.50 (38.00‐72.00)	.029
rMSSD (ms)	25.00 (17.00‐32.00)	30.50 (19.00‐43.00)	.248
pNN50 (%)	19.00 (8.00‐87.00)	75.50 (23.00‐122.00)	.294
BB50 (beats)	3340.50 (1045.00‐8406.00)	4002.00 (1920.00‐6561.00)	.729
Frequency domain:			
VLF (ms)	22.72 (18.57‐26.15)	35.99 (20.03‐45.60)	.020
LF (ms)	13.89 (11.06‐18.54)	24.09 (14.94‐33.82)	.006
HF (ms)	8.72 (6.11‐13.46)	12.88 (8.82‐16.46)	.034
LF/HF ratio	1.59 (1.19‐2.03)	1.79 (1.53‐2.44)	.181

Note

Data are presented as median (IQR).

Abbreviations: ASDNN, mean of five‐minute standard deviations of intervals; BB50, count of intervals that are more than 50 ms different from the previous interval; HF, high‐frequency power; HTN, hypertension; LF, low‐frequency power; mean NN, average of all intervals between normal beats excluding ectopy or noise intervals; pNN50, percentage of intervals that are more than 50 ms different from the previous interval; rMSSD, root mean square of the difference of successive R‐R intervals; SDANN, standard deviation of five‐minute mean R‐R interval; SDNN, standard deviation of intervals of all normal beat.

### 3.5 Multivariate adjusted factors related to uncontrolled blood pressure of hypertensive patients

Multiple regression analysis showed that HRV parameters: SDNN, SDANN, and rMSSD values were independent risk factors of uncontrolled blood pressure in hypertensive patients after adjusting for age, sex, BMI, amiodarone use, beta‐blockers use, ACE‐i/ARB use, CCB use, and diuretic use (Table 6).

**TABLE 6:** Multivariate logistic regression for risk of uncontrolled blood pressure in hypertensive group

	B	S.E	Wald	*P*‐value	Exp (B)	95% CI lower limit	95% CI upper limit
Sex (male)	−1.812	0.893	4.122	.042	0.163	0.028	0.939
BMI	0.330	0.133	6.112	.013	1.391	1.071	1.807
SDNN	−0.095	0.039	5.838	.016	0.909	0.842	0.982
SDANN	0.056	0.028	4.041	.044	1.058	1.001	1.117
rMSSD	0.211	0.096	4.836	.028	1.235	1.023	1.490
HF	−0.300	0.165	3.323	.068	0.741	0.537	1.023

Abbreviations: BMI, body mass index; HF, high‐frequency power; rMSSD, root mean square of the difference of successive R‐R intervals; SDANN, standard deviation of five‐minute mean R‐R interval; SDNN, standard deviation of intervals of all normal beat.

## 4 DISCUSSION

This study showed that decreased HRV level has significant association with increased BP. This significance is independent in every confounder tested by both frequency and time domain. These results are in‐line with previous findings for Asian population which showed that impaired autonomic nervous function in hypertensive patients is strongly associated with uncontrolled BP.^7^, ^9^, ^10^, ^11^, ^12^ Our study showed that hypertensive patients had significantly lower SDNN (reflecting vagal function)^10^ as well as meanNN, ASDNN, rMSSD (reflecting vagal function),^7^ pNN50 (reflecting vagal function),^7^ and BB50, VLF (reflecting vagal function), HF (reflecting vagal function),^7^ and LF/HF ratio (reflecting sympathovagal balance)^13^ compared to non‐hypertensive patients. SDNN, SDANN, ASDNN, VLF, LF (reflecting sympathovagal balance),^7^ and HF were significantly lower in hypertensive group with uncontrolled BP. Our result revealed that hypertensive patient had greater impairement in cardiac autonomic nervous activity than non‐hypertensive patients. More severe impairment was showed in hypertensive group with uncontrolled BP.

Multiple studies investigating the relationship between HRV and HTN were done in Asian population. Khoicybekov et al used five‐minute ECG recording to calculate non‐linear indices D2, K2, and lagged Poincaré plot. The data reported that heart rhythm variability in HTN group was less in variability than in non‐HTN group, which was expressed in lower entropy and correlation dimension.^12^ A similar study in India involving 30 hypertensive patients and 30 non‐hypertensive patients showed significantly reduced HFnu, SDNN, rMSSD, pNN50, and significantly increased LFnu and LF‐HF ratio in hypertensive individuals. The study also used five‐minutes of ECG recording.^10^ A study from Japan showed parasympathetic nervous system activity impairment were associated with increased ambulatory mean arterial pressure in the morning.^11^ Another study suggested that HRV reflects diastolic BP better than systolic BP levels and that alcohol intake strongly affected systolic BP levels in men, which may had weakened the association with HRV.^9^ In Chinese population, a study showed that reduced HRV and HRT were present in hypertensive patients, particularly in hypertensive patients with uncontrolled BP.^7^

HRV can be used to evaluate cardiac autonomic activity. It shows us the oscillation of heart rate which reflect the sympathetic and vagal function that regulates the heart rate response to any stimuli.^7^, ^14^, ^15^ Apart from external stimuli, HRV is also affected by internal stimuli, including circadian rhythms, core body temperature, metabolism, the sleep cycle, and the renin‐angiotensin system. Using 24‐hour HRV recordings is the “gold standard” of clinical HRV assessment because it provides greater predictive power than short‐term measurements.^16^ Deviation of HRV from the normal range is associated with various cardiovascular diseases. The 24‐hour recording of SDNN is the "gold standard" for medical stratification of cardiac risk. The SDNN value below 50 ms, 50‐100, and above 100 are classified as unhealthy, compromised health, and healthy, respectively.^16^

Essential hypertension results from an increase in systemic vascular resistance, which is greatly provoked by enhanced activity of sympathetic nervous system. Baroreceptor resetting, norepinephrine spillover, increased angiotensin II level in circulation, and local factors like endothelin lead to sympathetic hyperactivity.^17^, ^18^ This sympathetic hyperactivity may ultimately induce sympathovagal imbalance and decreased HRV in hypertensive patients.^10^

Some studies reported the effect of BP medication, including beta‐blocker, ACE inhibitor, ARB, and diuretic to HRV. Beta‐blockers users had equal or greater HRV than non‐users, whereas those using diuretics or ACE inhibitors had a lower HRV.^14^ Captopril medication increased HRV expressed as total power and LF power in the frequency domain.^19^ Anti‐arrhythmic drug amiodarone also affected HRV. Amiodarone administration showed a reduction of pNN50 and rMSSD.^20^ In this study, we performed multivariate regression to confounding factors, including anti‐arrhythmic and blood pressure medication use.It showed a significant association between several HRV parameters and hypertension, particularly in hypertensive patients with uncontrolled BP. There was considerable effect modification by antihypertensive medication use, with stronger associations among individuals not using antihypertensive medications.

### 4.1 Clinical implication and recommendation

Monitoring HRV, which reflects the cardiac sympathetic and vagal function, can be useful to evaluate the autonomic nervous function status of hypertensive patients and optimize therapeutic efficacy to improve autonomic nervous function balance. Moreover this study suggests that a prospective study is needed to find the casual relationship between decreased autonomic nervous function and new‐onset HTN or cardiovascular disease, especially in Asian population. HRV might also be able to predict the future risk of HTN at an earlier stage and prognosis during treatment.

### 4.2 Study limitation

Several limitations of this study should be considered. First, we could not confirm the presence of a causal relationship between cardiac autonomic nervous impairment and HTN due to the cross‐sectional design of the study with a relatively small sample size. Second, age of patients in the HTN group was significantly higher than those in the non‐HTN group. HRV parameters in an elderly population are usually lower than those of a younger population; therefore, this result is possibly biased in terms of age. After adjusting for age as one of risk factors of HTN in multivariate model, we found that HRV is an independent risk factor for HTN. Third, we could not exclude the effects of medication that affect autonomic cardiac function. Fourth, most subjects recruited in this study were patients with arrhythmia indicated for Holter study. This might cause population bias, and the result of this study only represent a population of patient with arrhythmia and HTN.

## 5 CONCLUSION

Our study showed that cardiac autonomic nervous impairment, as demonstrated by reduced HRV, is significantly associated with HTN. Decreased HRV was also significantly associated with uncontrolled blood pressure in hypertensive patients.

## ETHICAL APPROVAL AND CONSENT TO PARTICIPATE

Dr Soetomo General Hospital conferred ethical clearance for this study (Ref: 1822/KEPK/II/2020; February 20, 2020). A written informed consent was obtained from all patients.

## CONFLICT OF INTEREST

Authors declare no conflict of interests for this article.

## Supporting information

Table S1‐S2
