# Supplementary material for: Evaluation of heart rate variability using 24‐hour Holter electrocardiography in hypertensive patients
Source: J Arrhythm. 2020 Nov 28;37(1):157–64. doi: 10.1002/joa3.12469 (PMC7896473; doi:10.1002/joa3.12469)
Supplement: Supplementary file 1 — Table S1‐S2 [file JOA3-37-157-s001.docx]

**Supplementary table 1-2**

**Supplementary table 1.** Area Under the Curve (AUC) for the prediction of hypertension by SDNN, ASDNN, rMSSD, pNN50, BB50, VLF, and HF.

| **Area Under the Curve** | | | | | |
| --- | --- | --- | --- | --- | --- |
| Test Result Variable(s) | Area | Std. Error^a^ | Asymptotic Sig.^b^ | Asymptotic 95% Confidence Interval | |
|  |  |  |  | Lower Bound | Upper Bound |
| SDNN | ,626 | ,054 | ,024 | ,520 | ,732 |
| ASDNN | ,631 | ,054 | ,020 | ,525 | ,737 |
| rMSSD | ,621 | ,054 | ,031 | ,515 | ,728 |
| pNN50 | ,646 | ,053 | ,009 | ,542 | ,750 |
| BB50 | ,668 | ,052 | ,003 | ,567 | ,769 |
| VLF | ,619 | ,055 | ,034 | ,512 | ,726 |
| HF | ,634 | ,054 | ,017 | ,529 | ,739 |
| The test result variable(s): SDNN, ASDNN, rMSSD, pNN50, HF has at least one tie between the positive actual state group and the negative actual state group. Statistics may be biased. | | | | | |
| a. Under the nonparametric assumption | | | | | |
| b. Null hypothesis: true area = 0.5 | | | | | |

**Supplementary table 2.** Area Under the Curve (AUC) for the prediction of uncontrolled blood pressure in hypertensive group by SDNN, SDANN, ASDNN, VLF, LF, and HF.

| **Area Under the Curve** | | | | | |
| --- | --- | --- | --- | --- | --- |
| Test Result Variable(s) | Area | Std. Error^a^ | Asymptotic Sig.^b^ | Asymptotic 95% Confidence Interval | |
|  |  |  |  | Lower Bound | Upper Bound |
| SDNN | ,672 | ,081 | ,043 | ,513 | ,831 |
| SDANN | ,673 | ,081 | ,041 | ,514 | ,832 |
| ASDNN | ,685 | ,084 | ,029 | ,520 | ,851 |
| VLF | ,698 | ,085 | ,020 | ,532 | ,863 |
| LF | ,735 | ,079 | ,006 | ,581 | ,890 |
| HF | ,680 | ,082 | ,034 | ,519 | ,840 |
| The test result variable(s): SDNN, ASDNN has at least one tie between the positive actual state group and the negative actual state group. Statistics may be biased. | | | | | |
| a. Under the nonparametric assumption | | | | | |
| b. Null hypothesis: true area = 0.5 | | | | | |
